# Kinesiophobia and Psychological Readiness of Return to Sport in High-Performance Judokas After an Injury: A Cross-Sectional Study

**DOI:** 10.3390/medicina62030587

**Published:** 2026-03-20

**Authors:** Ulises Puchalt-Muñoz, Mireia Yeste-Fabregat, Helio Carratalá-Bellod, Marta Martínez-Soler, Rómulo J. González-García, Juan Vicente-Mampel

**Affiliations:** 1 Department of Physiotherapy, Medicine and Health Sciences School, Catholic University of Valencia, 46001 València, Spain; 2 Department of Physical Activity and Sports Sciences, Faculty of Physical Activity and Sport Sciences, Catholic University of Valencia, 46001 València, Spain; helio.carratala@ucv.es; 3 Campus Capacitas, Catholic University of Valencia, 46001 València, Spain

**Keywords:** judo, injury, kinesiophobia, self-perceived readiness to return to sport, readiness, return to sport

## Abstract

*Background and Objectives*: Judo is an Olympic contact sport with a high risk of injury owing to its physical, technical, and competitive demands. The role of psychological factors in recovery and Return to Sport (RTS), such as kinesiophobia and self-perception, is key in the injury process. These factors influence both the success and timing of return and are affected by variables such as locus of control, previous experience, and contextual factors. This study sought to analyse the relationship between sociodemographic, clinical, sports, and psychological variables with kinesiophobia and self-perception of RTS to identify psychological profiles. *Materials and Methods*: A cross-sectional observational study was conducted at the Centro de Alto Rendimiento de Judo (CEAR) in Valencia, Spain; involving 51 high-performance judokas (mean age 23.0 ± 3.8 years) competing at national or international level who were injured, out of competition or in the process of returning to training or competition. Data were collected using a self-administered questionnaire. Psychological variables were assessed using the Tampa Scale for Kinesiophobia (TSK-11) and the Psychological Readiness of Injured Athlete to Return to Sport (PRIA-RS) questionnaire. *Results*: No significant associations were found between sociodemographic, clinical–sports, and psychological variables (*p* > 0.05). The mean TSK-11 and PRIA-RS scores were 25.02 ± 5.79 and 36.49 ± 5.29, respectively. Cluster analysis identified three differentiated psychological profiles: one with high kinesiophobia, longer injury and time away from competition, and lower self-perceived readiness to RTS (*n* = 16); a second with lower fear, the lowest readiness, younger age, and shorter recovery time (*n* = 17); and a third with the lowest kinesiophobia, highest readiness, older age, and intermediate injury-related time (*n* = 18). *Conclusions*: Three psychological profiles were identified: young judokas with low self-perceived readiness to Return to Sport (RTS) and low kinesiophobia; older judokas with high readiness and minimal kinesiophobia; and a more vulnerable group with longer recovery times, high kinesiophobia, and low self-perceived readiness to RTS. Further studies with additional specific variables and biopsychosocial approaches are needed.

## 1. Introduction

Judo is an Olympic combat sport combining standing and groundwork techniques, requiring physical capacities such as maximal strength, endurance, power, flexibility, and aerobic and anaerobic conditioning [1,2,3], along with complex technical and tactical skills [4,5]. Its dynamic, high-contact nature leads to a significant injury risk, comparable to or higher than in team sports [6,7], with reported incidence ranging from 11 to 12% in Olympic competitions [8] to 1.18 injuries per athlete per year [9]. Most injuries are mild, though 24.4% require ≥8 treatment days [6], with the knee and shoulder being most affected [8,10]. Heavier female athletes often sustain more severe injuries, while lightweight athletes experience milder injuries, a pattern possibly linked to Rapid Weight Loss (RWL) practices [6], which affect up to 96% of athletes [11] and may cause dehydration, performance decline, and psychological effects such as tension, fatigue, irritability, sleep disturbances, and disordered eating [12]. Early RWL may predispose athletes to chronic stress, emotional dysregulation, and eating disorders [11].

Judo injuries affect not only athletes’ physical well-being but also have a significant psychological and social impact [13,14]. The return-to-sport process involves multiple physical, psychological, and contextual factors, such as competition demands, environmental conditions, or external pressure; making it a difficult and highly relevant decision [15]. Kinesiophobia is a common post-injury response, initially expressed as fear of movement leading to avoidance and, near the RTS phase, as fear of reinjury [16,17]. This fear–avoidance cycle promotes disuse and muscle inhibition, reinforcing the perception of disability and ultimately impairing physical function and self-confidence [18]. Athletes who are not psychologically ready to RTS display stronger fear–avoidance behaviours and adopt protective or hypervigilant attitudes, which can negatively affect performance [19]. In addition, returning before adequate psychological readiness increases fear, anxiety, and performance decline [19], and higher fear–avoidance levels are associated with poorer functional outcomes [20].

Therefore, addressing fear of movement during rehabilitation is essential. Ardern et al. emphasized that factors such as motivation, confidence, and the absence of fear are associated with a faster and more successful return [21]. Moreover, athletes’ self-perceived competence has been shown to directly influence RTS outcomes, as believing in one’s ability to meet performance demands is crucial for a confident and sustainable return to competition [22]. In judo, athletes’ self-perceived readiness plays decisive roles in Return to Sport (RTS) outcomes. For instance, recent research in judo athletes reported that 55.9% returned to sport after injury, whereas 44.1% did not resume sport participation [23]. Judokas who successfully return to training and competition report significantly higher levels of psychological readiness, and self-perceived psychological status has been identified as a significant predictor of RTS, even after accounting for clinical and injury-related factors [23]. Recent evidence highlights the clinical relevance of assessing psychological readiness during the RTS process. A recent systematic review reported that self-reported measures of psychological readiness are associated with return-to-sport outcomes, reinjury risk, and post-return performance and emphasised the value of multidimensional assessments integrating cognitive, emotional, and behavioural components. These findings support the use of comprehensive self-reported instruments to evaluate psychological readiness in injured athletes [24].

Several psychological factors influence kinesiophobia and self-perceived readiness to return to sport (RTS). Locus of control, or the extent to which individuals perceive outcomes as determined by their own actions (internal) versus external factors such as luck, affects recovery: an internal locus promotes self-regulation and responsibility, while an external locus is linked to greater vulnerability [25,26]. Identification with other injured athletes can either support injury prevention or increase fear, avoidance, and negative self-perception [27,28]. Fear of reinjury and kinesiophobia can delay rehabilitation and reduce the likelihood of returning to pre-injury performance, with the intensity of these responses varying according to injury severity and characteristics. Despite growing recognition of the psychological dimension of injury, research on high-performance judo remains limited, with most studies focusing on physical factors. Understanding the relationship between kinesiophobia and psychological readiness may inform more effective and safer rehabilitation strategies. This study aimed to analyse sociodemographic, clinical, and psychological determinants of kinesiophobia and self-perceived readiness in injured competitive judokas, while also reporting injury incidence and characteristics and identifying psychological profiles associated with RTS. We hypothesised that higher kinesiophobia would be associated with longer recovery times and lower perceived readiness in high-performance judokas.

## 2. Materials and Methods

### 2.1. Study Design

A cross-sectional, descriptive, quantitative study was conducted to analyse the presence of psychological variables influencing high-performance judokas during the injury process. This study was approved by the Ethics Committee of the Catholic University of Valencia (reference number: UCV/2022-2023/082). All participants voluntarily and anonymously agreed to participate by providing written or electronic informed consent prior to participation, in accordance with the ethical principles of the Declaration of Helsinki [29]. The trial adhered to the STROBE (Strengthening the Reporting of Observational Studies in Epidemiology) guidelines in both its design and the progression of participants [30].

### 2.2. Participants and Settings

The study included 51 high-performance judokas from the Centro de Alto Rendimiento de Judo (CEAR) of Valencia, Spain, all of whom had participated in national or/and international competitions. Recruitment and data collection took place on-site at the CEAR facilities in April 2025. All participants were informed of the data collection procedures. This research was conducted within the framework of a project led and supervised by the UCV. The inclusion criteria were as follows: (i) active male and female high-performance judokas (≥16 years old); (ii) Spanish-speaking athletes; (iii) having suffered a relevant musculoskeletal injury during judo practice; (iv) currently out of competition or in the process of returning to training or competition (RTS); and (v) voluntary acceptance of informed consent. The exclusion criteria were as follows: (i) incomplete questionnaires; (ii) chronic sequelae that had previously prevented the athlete from continuing professional sports practice; (iii) current pain intensity ≥ 4 on the Visual Analogue Scale (VAS); and (iv) time since injury <2 weeks at the time of assessment.

### 2.3. Sample Size

The sample size was considered appropriate for a cross-sectional descriptive and analytical study in sports psychology and injury perception, given the exploratory nature of the research and limited accessibility of elite athletic populations. Comparable sample sizes have been reported in previous cross-sectional studies, including those by Borsati et al., who examined exercise perception in samples of approximately 50 participants, and Khalil et al., who identified robust clinical and demographic associations in a cohort of 30 participants who underwent meniscal procedures [31,32]. Participants were recruited through a non-probabilistic convenience sampling strategy, commonly used in high-performance athletes due to their limited accessibility. The sample consisted exclusively of national and international-level competitors, enhancing the specificity and contextual relevance of the findings. No a priori sample size calculation was performed due to the exploratory nature of the study and the limited accessibility of elite athletic populations.

### 2.4. Measurement Variable

Data were collected at a single time point without follow-up or therapeutic intervention. Participants completed an online self-administered questionnaire under supervision to ensure consistency and high data quality. The questionnaire included sociodemographic and clinical–sport variables and incorporated two validated instruments to assess psychological variables.

#### 2.4.1. Sociodemographic and Clinical-Sport Variables

Sex and age were sociodemographic variables. The clinical and sports variables included competitive weight category, according to the International Judo Federation (IJF), injury type (ligament, muscle, fracture, contusion, or dislocation), injury location (by anatomical region), time since injury onset (in months), and time out of competition (in months).

#### 2.4.2. Tampa Scale for Kinesiophobia: 11 Items Version (TSK-11)

Fear of movement was assessed using the TSK self-administered questionnaire. The instrument consists of 11 items, each rated on a 4-point Likert scale ranging from “strongly disagree” to “strongly agree” The total score is interpreted as a continuous index, with higher values indicating a greater degree of kinesiophobia. Although the TSK-11 is commonly interpreted using a total score, factorial analyses of the Spanish version have identified a stable two-factor structure comprising Activity Avoidance (AA) and Harm (H). The Spanish version, validated for the Spanish-speaking population in 2011, demonstrated adequate psychometric properties in patients with both acute and chronic pain, confirming a stable two-factor structure and showing good internal consistency (Cronbach’s α = 0.79 for the total score; α = 0.79 for AA; α = 0.70 for H), as well as evidence of convergent validity through significant associations with pain-related psychological variables and predictive validity over time [33]. The TSK-11 has proven to be a widely used tool across multiple populations, and its short version reduces respondent burden without compromising measurement quality, making it particularly suitable for athletic populations owing to its reliability and accessibility [34,35].

#### 2.4.3. Psychological Readiness of Injured Athlete to Return to Sport Questionnaire (PRIA-RS)

Self-perceived psychological readiness for RTS was assessed using PRIA-RS in its original Spanish version. The instrument consists of 10 items rated on a 5-point Likert scale (total score 10–50), with higher values indicating greater readiness. Scores ≥ 40 reflect adequate readiness, 35–39 suggest the need for further assessment, and <35 indicate inadequate readiness. This questionnaire was developed and validated in Spanish using the Delphi method, with high content validity (Aiken’s V > 0.90) [36]. Additionally, the questionnaire has been translated and culturally adapted into other languages, including English, Greek, and Turkish, further supporting its international applicability [37,38,39].

It was inspired by recognised scales such as the TSK, Anterior Cruciate Ligament–Return to Sport after Injury (ACL-RSI) and Injury-Psychological Readiness to Return to Sport (I-PRRS), addressing key components including coping, confidence, emotional state, external pressure, and perceived security [36]. Its psychometric performance has also been confirmed in later analyses of injured athletes [40].

### 2.5. Statistical Analysis

Categorical variables were expressed as frequencies and percentages, while continuous variables with a normal distribution were summarized using means, standard deviations (SD), interquartile ranges, and 95% confidence intervals (CIs). Normality of continuous variables was assessed using the Shapiro–Wilk test and visual inspection of Q–Q plots. Relationships between study variables were examined using inferential and correlation analyses. Specifically, independent-sample Student’s *t*-tests were used to analyse differences by sex, and one-way ANOVA tests were performed to examine differences according to injury type, injury location, and weight category. To assess the relationship between TSK-11 scores and PRIA-RS categories, a one-way ANOVA was conducted to compare kinesiophobia across readiness groups defined by the questionnaire. Effect sizes were calculated to quantify the magnitude of group differences, using Cohen’s d for *t*-tests and eta squared (η^2^) for ANOVA, together with their corresponding 95% confidence intervals. The effect size (ES) was estimated by calculating Cohen’s d coefficient. ES was classified as trivial (<0.20), small (0.20–0.49), moderate (0.50–0.79), or large (>0.80). Pearson’s correlation coefficients were calculated to explore associations between continuous variables, including age, time since injury onset, time out of competition, and psychological scores (TSK-11 and self-perceived readiness for return to sport). Additionally, a K-means cluster analysis was conducted to identify psychological and clinical profiles based on standardized quantitative variables, including age, time since injury onset, time out of competition, and psychological scores from both questionnaires. Solutions ranging from two to seven clusters were compared using the Silhouette Index, and the configuration with the highest internal cohesion and clinical interpretability was selected. Statistical significance was set at *p* < 0.05. All analyses were performed using JAMOVI software (version 2.6.26.0.) [41].

## 3. Results

### 3.1. Sample Characteristics

**Figure 1:**
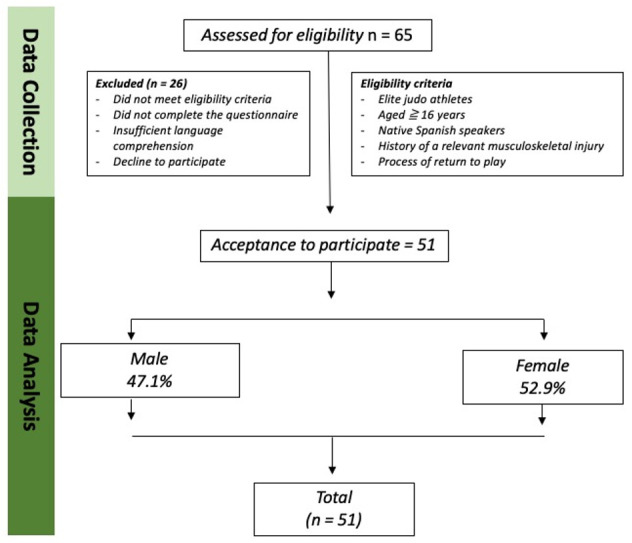
Flow Chart Participants.

Figure 1 provides comprehensive details regarding the recruitment and progression of participants. All 51 participants completed the questionnaire, resulting in no missing data.

The study sample comprised Spanish-speaking judokas competing at national or international levels, all aged over 16 years, including 27 women (52.9%) and 24 men (47.1%), with a mean age of 23.04 ± 3.79 years. Table 1 presents a summary of the descriptive sociodemographic and clinical-sport characteristics of the sample. This summary includes participants age, sex, weight category, injury location and type, time since injury onset and time out of competition.

### 3.2. Psychological Variables

Descriptive statistics and sex comparison of psychological variables are presented in Table 2. The assumption of homogeneity of variances was met in all analyses, as confirmed by Levene’s test (*p* > 0.05). No statistically significant differences were observed between sexes for any psychological variable (*p* > 0.05).

### 3.3. Inferential Analysis

Prior to group comparisons by weight category, the <100 kg and >100 kg male categories were merged to ensure adequate sample sizes, as statistical analyses cannot be performed on categories containing only one participant. Subsequently, a series of one-way ANOVA tests was conducted to examine differences in psychological scores according to weight category (for both sexes), injury location, injury type, and psychological readiness categories defined by the PRIA-RS. The assumption of homogeneity of variances was met in all analyses, as confirmed by Levene’s test (*p* > 0.05). No statistically significant differences were observed across groups (*p* > 0.05). ANOVA comparisons are summarized in Table 3.

Pearson correlation analyses were performed to explore linear associations between age, time since injury onset, time out of competition, and psychological variables. No significant associations were identified (*p* > 0.05), except for strong positive correlations between the TSK-11 subscales and the total TSK-11 score, confirming the internal consistency of the scale. A positive association was observed between time since injury and time out of competition, showing trend toward statistical significance (r = 0.27, *p* = 0.055). No other significant associations were detected between TSK-11 scores and PRIA-RS scores. The correlation matrix is presented in Table 4.

### 3.4. Cluster Analysis

**Figure 2:**
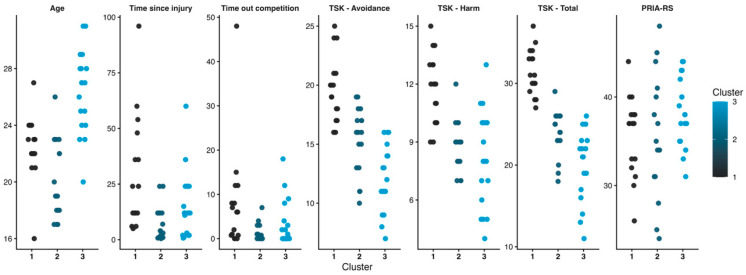
Distribution of participants across clusters across study variables.

Although the four-cluster solution yielded a higher Silhouette Index (SI = 0.529), it resulted in a single-case cluster and was therefore not retained. Instead, the three-cluster solution was selected as it provided the best balance between internal cohesion and clinical interpretability (SI = 0.471). The three clusters comprised 16, 17, and 18 participants, respectively, and revealed distinct psychological and clinical profiles. Cluster 1 was characterized by elevated levels of kinesiophobia across both TSK-11 subscales, a longer duration since injury onset, extended absence from competition, and slightly below-average self-perceived readiness to RTS scores on the PRIA-RS. Cluster 2 exhibited intermediate kinesiophobia, the lowest PRIA-RS scores, younger age, and shorter durations since injury and competition absence. Cluster 3 demonstrated the lowest kinesiophobia levels, the highest PRIA-RS scores, older age, and near-average values for injury duration and time away from competition. The distribution of participants across clusters is illustrated in Figure 2, while standardized cluster centroids (z-scores) are presented in Table 5.

## 4. Discussion

The overall sample homogeneity, particularly regarding sex and age, provided a balanced context for comparison. Knee and shoulder injuries were the most prevalent, in line with previous epidemiological data [8], although the predominance of ligamentous and muscular injuries differs from reports describing higher rates of sprains, fractures, and dislocations [10]. No significant differences were found in psychological variables according to sex, weight category, injury characteristics, or self-perceived readiness to RTS groups, nor were relevant correlations observed with age or injury-related timelines, except for strong associations within the TSK-11. Injury timelines showed the expected wide variability, consistent with the inclusion of athletes with diverse injury types and severities. However, cluster analysis revealed three psychological profiles, indicating that person-centred approaches capture meaningful psychological variability not detected by traditional group-based analyses. These findings align with previous cluster-based studies in sports psychology that identified psychological profiles in athletes using self-reported constructs and considering differences in age and competitive experience [42,43].

The absence of sex and weight categories differences contrasts with studies reporting greater psychological vulnerability among female athletes, strongly linked to the widespread use of RWL practices, often poorly controlled and accompanied by negative emotional profiles [11,44]. Female judokas appear particularly vulnerable, showing greater emotional distress and body image dissatisfaction, as well as a high risk of developing eating disorders, with Rouveix et al. reporting that 91.7% were at significant risk [45]. Similarly, heavier weight categories, where RWL practices are generally less frequent, were expected to show more favourable psychological profiles; however, no differences emerged despite the documented negative effects of RWL [12]. Injuries within interdependent training environments may also disrupt team dynamics, decrease motivation, and foster an external locus of control [19].

Among contextual factors, competitive pressure, rapid decision-making under stress, sustained focus amid changing and aggressive stimuli, and the individual nature of performance outcomes intensify psychological demands [45,46]. The lack of significant differences in the present study may reflect the influence of unmeasured psychological factors that modulate individual emotional responses to injury. Previous studies have shown that coping styles mediate the relationship between resilience and perceived stress, modulating athletes’ emotional responses to injury, while both trait and state anxiety are associated with perceived stress, with this relationship moderated by cognitive reappraisal strategies [47,48]. Moreover, an internal locus of control and higher openness to experience personality trait have been associated with faster recovery and greater readiness to RTS [49]. These psychological responses may also be influenced by individual biological variability, potentially contributing to heterogeneous post-injury adaptation [46]. Overall, these factors may be key to athletes’ psychological adaptation after injuries.

Neither clinical nor sport-related variables showed significant correlations, which may reflect sample-specific factors (case selection, injury severity, timing of assessment) or differences in diagnostic or reporting criteria. Specifically, the lack of associations with injury-related timelines differs from previous evidence, which has suggested that positive psychological readiness facilitates RTS [21]. Instead, factors such as rehabilitation support, injury severity, and individual coping processes, particularly acceptance of the recovery process and lower catastrophizing, may exert a stronger influence on psychological adjustment and readiness to return [50]. The main psychological outcomes reflected an intermediate profile, with a mean TSK-11 score below the scale midpoint, indicating mild fear of movement without clinically relevant kinesiophobia. This pattern suggests a cautious but functional response and aligns with the findings in both combat and non-contact sports [51,52], with similar scores across athletic samples. Similarly, PRIA-RS scores indicated an intermediate self-perceived readiness to RTS, with only 19% of athletes showing adequate predisposition to return, while approximately 80% presented some degree of psychological vulnerability, highlighting the relevance of self-perception in the return-to-sport process. No association between TSK-11 and PRIA-RS further implies that kinesiophobia does not necessarily entail low confidence in RTS.

Cluster analysis offers a more nuanced characterisation of psychological profiles beyond group-based comparisons. The youngest athletes (Cluster 2) exhibited low kinesiophobia but reduced confidence in returning to sport, suggesting limited psychological maturity and less consolidated self-regulatory resources, likely influenced by fewer prior injury experiences. In contrast, older athletes (Cluster 3) showed low fear of movement alongside high self-perceived readiness, consistent with more effective emotional regulation and adaptive strategies, potentially associated with age-related maturity in sports and certainly shaped by previous recovery experiences. Finally, Cluster 1 presented high kinesiophobia, prolonged recovery times, and low self-perceived readiness to RTS. This profile may reflect the maladaptive fear–avoidance pattern described in the previous literature, where avoidance strategies progressively consolidate and may contribute to longer injury timelines. Specifically, this model proposes that when pain or injury-related sensations are interpreted as threatening, athletes may develop fear of movement, leading to protective avoidance behaviours. Furthermore, according to Pavlov’s and Skinner’s conditioning models, movements associated with pain can trigger an automatic fear response, which explains how avoiding painful movements temporarily reduces anxiety, reinforcing the fear–avoidance pattern and potentially delaying functional recovery and RTS, as avoidance behaviours become maladaptive strategies over time [53,54]. Moreover, the combination of elevated fear and reduced self-perceived readiness observed in this cluster may reflect greater psychological vulnerability during the recovery process [18]. Personality traits such as neuroticism or perfectionism may favour avoidant coping, while active coping and cognitive restructuring support better adaptation [55]. Finally, some authors have suggested that genetic differences may modulate psychological traits relevant to injury recovery [46].

### 4.1. Limitations

This study has several limitations that should be considered when interpreting the results. First, the psychological variables assessed captured only a part of the multidimensional return-to-sport process. The lack of detailed injury-related information, added to the absence of relevant contextual and RWL variables, may have constrained the analysis, as well as the absence of information about previous injuries and prior sports experience, as predicted by the literature [8]. Furthermore, the uneven distribution of some variables limited the representativeness, as not all injury types, locations, or weight categories were equally represented. In this context, the uneven distribution of some variables limited representativeness, and the weight category could not be included in the cluster analysis due to its nominal nature and low representation at certain levels. The cross-sectional design and reliance on self-reported questionnaires introduce potential sources of bias. Selection bias may be present due to voluntary participation, possibly favouring athletes with greater awareness of their rehabilitation process. Information bias and social desirability bias cannot be excluded, particularly in psychological variables, despite assurances of anonymity and supervised data collection. Moreover, as only Spanish-speaking athletes were included to ensure instrument validity, the generalisability of the findings to other linguistic and cultural contexts may be limited.

### 4.2. Future Research

Future research should incorporate more psychological constructs such as coping strategies, anxiety, locus of control, personality traits, injury acceptance, and catastrophizing, along with completed injury assessments and considering athletes’ previous injury and athletic maturity. Additionally, contextual factors including training environment, the influence of rapid weight loss practices, and social support should be considered or assessed. Longitudinal studies are needed to better understand how psychological readiness changes throughout the injury and recovery process and to assess the effects of multidisciplinary rehabilitation approaches. Finally, future research may benefit from adopting enactive perspectives of injury recovery, acknowledging that psychological responses are shaped through ongoing interactions between the athlete and their environment, including rehabilitation contexts, social dynamics, and sport-specific demands [56].

### 4.3. Practical Applications

The findings of this study may have several practical implications for clinicians, physiotherapists, sport psychologists, and coaches involved in the rehabilitation and return-to-sport (RTS) process of high-performance judokas.

First, the identification of different psychological profiles suggests that injured athletes should not necessarily be managed through a single standardized rehabilitation approach. Incorporating brief psychological screening tools such as the Tampa Scale for Kinesiophobia (TSK-11) and the Psychological Readiness of Injured Athlete to Return to Sport (PRIA-RS) questionnaire into routine assessments may help professionals identify athletes who could benefit from additional psychological support during rehabilitation. Early recognition of fear of movement or reduced psychological readiness may allow clinicians to adapt rehabilitation strategies more individually.

Second, athletes presenting with higher levels of kinesiophobia and longer recovery periods may particularly benefit from integrating psychological strategies into the rehabilitation process. Approaches such as progressive exposure to sport-specific movements, reassurance and education about the injury and recovery process, as well as gradual return-to-training protocols, may help reduce fear–avoidance behaviours and increase confidence in movement.

Third, the profile observed in younger athletes—characterized by relatively low kinesiophobia but lower perceived readiness to return to sport—suggests the possible influence of psychological maturity and previous injury experience in the recovery process. In this context, intervention strategies including educational guidance, confidence-building strategies, and structured decision-making during the RTS phase may help improve athletes’ perceived competence and readiness to resume sport participation. In addition, the literature on mental preparation in combat sports highlights the role of psychological skills training aimed at enhancing athletes’ confidence, stress regulation, anxiety management, and attentional control. Techniques such as visualization, inner speech regulation, mindfulness-based strategies, and relaxation training have been proposed as useful tools to support athletes’ mental preparation in demanding sport situations [57].

Finally, the results reinforce the importance of adopting a multidisciplinary approach in the rehabilitation of high-performance judokas. Combining physical rehabilitation with psychological monitoring may support more informed return-to-sport decisions, potentially contributing to safer reintegration into training and competition while reducing the risk of reinjury and supporting long-term performance.

From a practical perspective, the psychological profiles identified through cluster analysis may help clinicians adapt rehabilitation strategies according to the athletes’ psychological characteristics. Athletes belonging to the cluster with higher kinesiophobia and longer recovery periods may require specific interventions aimed at reducing fear of movement, such as gradual exposure to sport-specific tasks and reassurance during rehabilitation. In contrast, younger athletes with lower levels of kinesiophobia but reduced perceived readiness to return to sport may benefit from strategies focused on improving confidence, providing education about the recovery process, and offering structured guidance during the return-to-sport phase. Considering these psychological profiles during rehabilitation may help physiotherapists better individualize treatment and support safer return-to-sport decisions.

## 5. Conclusions

This study provides an overview of musculoskeletal injuries and psychological responses in high-performance judokas. No significant associations were found between sociodemographic or clinical-sport variables and psychological responses. Overall, athletes presented non-disabling levels of kinesiophobia but reduced self-perceived readiness to return to sport (RTS), with a high proportion reporting low readiness. Cluster analysis identified three distinct psychological profiles differing in age, recovery time, kinesiophobia, and self-perceived readiness to RTS. These findings highlight the importance of considering psychological factors during rehabilitation and return-to-sport decision-making in high-level judokas and support the need for further research examining how experience and injury-related factors influence recovery and RTS outcomes.

## Figures and Tables

**Table 1:** Descriptive analysis of sociodemographic and clinical-sport variables.

	Women		Men		Total
n	27 (52.9%)		24 (47.1%)		51
Age (years)	23.0 ± 3.73		23.1 ± 3.94		23.04 ± 3.79
Weight Category	+78 kg	3 (5.8%)	+100 kg	5 (9.8%)	
	−78 kg	2 (3.8%)	−100 kg	1 (2%)	
	−70 kg	5 (9.6%)	−90 kg	2 (3.9%)	
	−63 kg	4 (7.7%)	−81 kg	6 (11.8%)	
	−57 kg	3 (5.8%)	−73 kg	4 (7.8%)	
	−52 kg	5 (9.6%)	−66 kg	4 (7.8%)	
	−48 kg	5 (9.6%)	−60 kg	2 (3.9%)	
Injury location					
Knee	11 (21.6%)		10 (19.6%)		21 (41.2%)
Shoulder	9 (17.6%)		5 (9.8%)		14 (27.5%)
Elbow	4 (7.8%)		1 (2%)		5 (9.8%)
Wrist-hand	1 (2%)		3 (5.9%)		4 (7.8%)
Ankle-foot	1 (2%)		4 (7.8%)		5 (9.8%)
Lumbar spine	1 (2%)		1 (2%)		2 (3.9%)
Injury type					
Ligament	12 (23.5%)		10 (19.6%)		22 (43.1%)
Muscular	7 (13.7%)		6 (11.8%)		13 (25.5%)
Dislocation	6 (11.8%)		6 (11.8%)		12 (23.5%)
Contusion	2 (3.9%)		2 (3.9%)		4 (7.8%)
Injury onset (months)	16.13 ± 16.29		17.84 ± 22.01		16.9 ± 19.0
Time Out	4.62 ± 9.84		3.42 ± 4.33		4.06 ± 7.7

Data are presented as mean ± SD: standard deviation, and categorical variables are expressed as n (frequency %).

**Table 2:** Descriptive analysis and sex comparison of psychological variables.

Psychological Variable	Women	Men	Total		Sex Comparison (*t*-Test)
	Value (Mean ± SD)			Range	
TSK_A	15.44 ± 4.47	15.67 ± 3.91	15.55 ± 4.17	7–25	−0.1949; [−0.60, 0.50]; 0.852 and −0.05
TSK_H	9.30 ± 2.63	9.67 ± 2.37	9.47 ± 2.49	4–15	−0.5349; [−0.70, 0.40]; 0.601 and −0.15
TSK_T	24.74 ± 6.02	25.33 ± 5.63	25.02 ± 5.79	11–37	−0.3649; [−0.65, 0.45]; 0.719 and −0.10
PRIA-RS	36.26 ± 6.16	36.75 ± 4.22	36.49 ± 5.29	24–48	−0.3349; [−0.64, 0.46]; 0.745 and −0.09
PRIA-RS: Not_ready	8 (15.7%)	10 (19.6%)	18 (35.3%)		
PRIA-RS: More_assess	14 (27.5%)	9 (17.6%)	23 (45.1%)		
PRIA-RS: Ready	5 (9.8%)	5 (9.8%)	10 (19.6%)		

Data are presented as mean ± SD: standard deviation, and categorical variables are expressed as n (frequency %). Sex comparisons were conducted using independent-sample *t*-tests and are reported as tdf; CI95% of d; p and d. t indicates the test statistic, df: degrees of freedom, CI95%: 95% confidence interval, p: significance *p*-value, d: Cohen’s d for independent *t*-tests. TSK: Tampa Scale of Kinesiophobia; TSK_A: TSK Avoidance subdimension, TSK_H: TSK Harm subdimension, TSK_T: TSK Total Score, PRIA-RS: Psychological Readiness of Injured Athlete to Return to Sport Questionnaire.

**Table 3:** ANOVA comparisons for psychological variables.

Psychological Variables	Female Weight Categories	Male Weight Categories	Injury Location	Injury Type	Self-Perceived Readiness PRIA-RS Categories
TSK_A	0.35620; 0.903 and 0.09	0.21518; 0.956 and 0.05	0.65545; 0.666 and 0.07	0.20347; 0.897 and 0.01	1.87248; 0.165 and 0.07
TSK_H	0.77620; [0.00, 0.42]; 0.601 and 0.19	0.60518; [0.00, 0.33]; 0.702 and 0.14	0.63545; [0.00, 0.17]; 0.677 and 0.07	1.08347; [0.00, 0.20]; 0.366 and 0.07	0.60248; [0.00, 0.13]; 0.555 and 0.02
TSK_T	0.63620; [0.00, 0.39]; 0.704 and 0.16	0.22518; [0.00, 0.22]; 0.950 and 0.06	0.59545; [0.00, 0.16]; 0.704 and 0.06	0.50347; [0.00, 0.15]; 0.682 and 0.03	1.65248; [0.00, 0.22]; 0.203 and 0.06
PRIA-RS	1.88620; [0.00, 0.58]; 0.134 and 0.36	0.23518; [0.00, 0.23]; 0.944 and 0.06	0.64545; [0.00, 0.17]; 0.674 and 0.07	2.58347; [0.00, 0.32]; 0.064 and 0.13	

Note: Values are reported as F, df1, df2; p and η^2^. F indicates the test statistic, df: degrees of freedom, p: significance *p*-value, and η^2^: eta squared for one-way ANOVA. TSK: Tampa Scale of Kinesiophobia; TSK_A: TSK Avoidance subdimension; TSK_H: TSK Harm subdimension; TSK_T: TSK Total Score; PRIA-RS: Psychological Readiness of Injured Athlete to Return to Sport Questionnaire.

**Table 4:** Pearson correlation matrix.

	Age	Time Since Injury	Time out Competition	TSK_A	TSK_H	TSK_T
Time since injury	0.0649; [−0.21, 0.33] and 0.652					
Time out competition	0.2549; [−0.02, 0.49] and 0.072	0.2749; [0.01, 0.51] and 0.055				
TSK_A	−0.1849; [−0.43, 0.10] and 0.214	0.2149; [−0.07, 0.46] and 0.134	0.1949; [−0.09, 0.44] and 0.180			
TSK_H	−0.0149; [−0.28, 0.27] and 0.977	0.2149; [−0.07, 0.46] and 0.140	0.1249; [−0.16, 0.39] and 0.388	0.4749; [0.23, 0.66] and <0.001 *		
TSK_T	−0.1349; [−0.39, 0.15] and 0.366	0.2449; [−0.03, 0.49] and 0.085	0.1949; [−0.09, 0.44] and 0.180	0.9249; [0.87, 0.96] and <0.001 *	0.7749; [0.63, 0.86] and <0.001 *	
PRIA-RS	0.1249; [−0.16, 0.38] and 0.415	−0.0949; [−0.36, 0.19] and 0.534	−0.1349; [−0.39, 0.15] and 0.378	−0.1549; [−0.40, 0.13] and 0.299	−0.0549; [−0.32, 0.23] and 0.713	−0.1349; [−0.39, 0.15] and 0.365

Note: Values are reported as df; CI95% of r and p. r indicates the Pearson’s correlation coefficient, df: degrees of freedom, CI95%: 95% confidence interval and p: significance *p*-value. Statistical significance is indicated as *p* < 0.001 (*). TSK: Tampa Scale of Kinesiophobia; TSK_A: TSK Avoidance subdimension; TSK_H: TSK Harm subdimension; TSK_T: TSK Total Score; PRIA-RS: Psychological Readiness of Injured Athlete to Return to Sport Questionnaire.

**Table 5:** Silhouette Index values and standardized cluster centroids for the three-cluster solution.

Silhouette Index						
Number of clusters	2	3	4	5	6	7
K-means cluster analysis (z-scores)	0.000	0.471	0.529	0.000	0.000	0.000
**K-means cluster analysis (z-scores)**						
	N	AGE	TSK AVOIDING	TSK HARM	TSK TOTAL	PRIA-RS_TOTAL
Cluster 1	16	−0.109	0.992	0.914	1.109	−0.152
Cluster 2	17	−0.832	−0.047	−0.307	−0.166	−0.226
Cluster 3	18	0.883	−0.837	−0.523	−0.829	0.348

Note: Silhouette Index values indicate the average Silhouette coefficient for each cluster solution; the selected solution is shown in bold. K-means cluster values were expressed as standardized scores (z-scores). TSK: Tampa Scale of Kinesiophobia; PRIA-RS: Psychological Readiness of Injured Athlete to Return to Sport Questionnaire.

## Data Availability

The data presented in this study are available on request from the corresponding author due to privacy and ethical restrictions.

## Abbreviations

The following abbreviations are used in this manuscript:

RTS	Return to Sport
CEAR	Centro de Alto Rendimiento de Judo
TSK-11	Tampa Scale for Kinesiophobia (11-item version)
PRIA-RS	Psychological Readiness of Injured Athlete to Return to Sport
STROBE	Strengthening the Reporting of Observational Studies in Epidemiology
IJF	International Judo Federation
RWL	Rapid Weight Loss
VAS	Visual Analogue Scale
ACL-RSI	Anterior Cruciate Ligament–Return to Sport after Injury scale
I-PRRS	Injury–Psychological Readiness to Return to Sport scale

## References

[1] Chaabene H., Negra Y., Bouguezzi R., Mkaouer B., Franchini E., Julio U., Hachana Y. (2017). Physical and Physiological Attributes of Wrestlers: An Update. J. Strength. Cond. Res..

[2] Karatrantou K., Katsoula C., Tsiakaras N., Ioakimidis P., Gerodimos V. (2020). Strength Training Induces Greater Increase in Handgrip Strength than Wrestling Training per Se. Int. J. Sports Med..

[3] Torres-Luque G., Hernández-García R., Escobar-Molina R., Garatachea N., Nikolaidis P.T. (2016). Physical and Physiological Characteristics of Judo Athletes: An Update. Sports.

[4] Branco B.H.M., Massuça L.M., Andreato L.V., Marinho B.F., Miarka B., Monteiro L., Franchini E. (2013). Association between the Rating Perceived Exertion, Heart Rate and Blood Lactate in Successive Judo Fights (Randori). Asian J. Sports Med..

[5] Franchini E., Del Vecchio F.B., Matsushigue K.A., Artioli G.G. (2011). Physiological Profiles of Elite Judo Athletes. Sports Med..

[6] Kim K.-S., Park K.J., Lee J., Kang B.Y. (2015). Injuries in National Olympic Level Judo Athletes: An Epidemiological Study. Br. J. Sports Med..

[7] Kujala U.M., Taimela S., Antti-Poika I., Orava S., Tuominen R., Myllynen P. (1995). Acute Injuries in Soccer, Ice Hockey, Volleyball, Basketball, Judo, and Karate: Analysis of National Registry Data. BMJ.

[8] Pocecco E., Ruedl G., Stankovic N., Sterkowicz S., Del Vecchio F.B., Gutiérrez-García C., Rousseau R., Wolf M., Kopp M., Miarka B. (2013). Injuries in Judo: A Systematic Literature Review Including Suggestions for Prevention. Br. J. Sports Med..

[9] Souza M., Monteiro H., Del Vecchio F., Gonçalves A. (2006). Referring to Judo’s Sports Injuries in São Paulo State Championship. Sci. Sports.

[10] Frey A., Lambert C., Vesselle B., Rousseau R., Dor F., Marquet L.A., Toussaint J.F., Crema M.D. (2019). Epidemiology of Judo-Related Injuries in 21 Seasons of Competitions in France: A Prospective Study of Relevant Traumatic Injuries. Orthop. J. Sports Med..

[11] Sundgot-Borgen J., Garthe I. (2011). Elite Athletes in Aesthetic and Olympic Weight-Class Sports and the Challenge of Body Weight and Body Compositions. J. Sports Sci..

[12] Štangar M., Štangar A., Shtyrba V., Cigić B., Benedik E. (2022). Rapid Weight Loss among Elite-Level Judo Athletes: Methods and Nutrition in Relation to Competition Performance. J. Int. Soc. Sports Nutr..

[13] Patenteu I., Gawrych R., Bratu M., Vasile L., Makarowski R., Bitang A., Nica S.A. (2024). The Role of Psychological Resilience and Aggression in Injury Prevention among Martial Arts Athletes. Front. Psychol..

[14] Lalayan G., Stepanyan L., Avetisyan A. (2023). An Investigation of Psychological and Physiological Factors Affecting Performance in Adolescent Judokas. Georgian Med. News.

[15] Meeuwisse W.H., Tyreman H., Hagel B., Emery C. (2007). A Dynamic Model of Etiology in Sport Injury: The Recursive Nature of Risk and Causation. Clin. J. Sport Med..

[16] Flanigan D.C., Everhart J.S., Pedroza A., Smith T., Kaeding C.C. (2013). Fear of Reinjury (Kinesiophobia) and Persistent Knee Symptoms Are Common Factors for Lack of Return to Sport After Anterior Cruciate Ligament Reconstruction. Arthrosc. J. Arthrosc. Relat. Surg..

[17] Khanna K., Jain S., Shetty G., Rahlan N., Ram C.S. (2022). Fear-Avoidance Beliefs, Kinesiophobia, and Disability Risk Among Indians with Spine Pain. Indian J. Orthop..

[18] Ambegaonkar J.P., Jordan M., Wiese K.R., Caswell S.V. (2024). Kinesiophobia in Injured Athletes: A Systematic Review. J. Funct. Morphol. Kinesiol..

[19] Juggath C., Naidoo R. (2024). The Influence of Psychological Readiness of Athletes When Returning to Sport after Injury. S. Afr. J. Sports Med..

[20] Fischerauer S.F., Talaei-Khoei M., Bexkens R., Ring D.C., Oh L.S., Vranceanu A.-M. (2018). What Is the Relationship of Fear Avoidance to Physical Function and Pain Intensity in Injured Athletes?. Clin. Orthop. Relat. Res..

[21] Ardern C.L., Taylor N.F., Feller J.A., Webster K.E. (2013). A Systematic Review of the Psychological Factors Associated with Returning to Sport Following Injury. Br. J. Sports Med..

[22] D’Astous E., Podlog L., Burns R., Newton M., Fawver B. (2020). Perceived Competence, Achievement Goals, and Return-To-Sport Outcomes: A Mediation Analysis. Int. J. Environ. Res. Public Health.

[23] Lambert C., Guenther D., Schütz L.-M., Kern N., Ritzmann R., Reinert N., Walz M., Wafaisade A., Nagy K., Reuter S. (2023). Psychological Readiness Is Related to Return to Sport in Judo Injuries: A Cross-Sectional Study. BMC Sports Sci. Med. Rehabil..

[24] Liu S., Noh Y.-E. (2025). The Utility of Psychological Readiness Scales in Predicting Return to Sport: A Systematic Review. BMC Psychol..

[25] Botha F., Dahmann S.C. (2024). Locus of Control, Self-Control, and Health Outcomes. SSM—Popul. Health.

[26] Murphy G.C., Foreman P.E., Simpson C.A., Molloy G.N., Molloy E.K. (1999). The Development of a Locus of Control Measure Predictive of Injured Athletes’ Adherence to Treatment. J. Sci. Med. Sport..

[27] Bandura A. (1986). Social Foundations of Thought and Action: A Social Cognitive Theory.

[28] Deroche T., Stephan Y., Woodman T., Le Scanff C. (2012). Psychological Mediators of the Sport Injury—Perceived Risk Relationship. Risk Anal..

[29] World Medical Association (2025). World Medical Association Declaration of Helsinki: Ethical Principles for Medical Research Involving Human Participants. JAMA.

[30] Cuschieri S. (2019). The STROBE Guidelines. Saudi J. Anaesth..

[31] Borsati A., Giannarelli D., Pase G., Ciurnelli C., Toniolo L., Trestini I., Tregnago D., Belluomini L., Sposito M., Insolda J. (2025). A Cross-Sectional Study Exploring the Perception of Exercise Oncology in the Italian Population. Front. Oncol..

[32] Khalil R.S.M., Mohamed A., Mohammed M.A.A., Elsiddig M., Rabah A.E.M.A., Abdallah R., Sovla H.M., Hussein S.H.M., Khalil R.S.M., Mohamed A. (2025). Analyzing the Relationship Between Various Factors and Their Influence on the Success Rates of Meniscal Injury Procedures: A Prospective Cross-Sectional Study. Cureus.

[33] Gómez-Pérez L., López-Martínez A.E., Ruiz-Párraga G.T. (2011). Psychometric Properties of the Spanish Version of the Tampa Scale for Kinesiophobia (TSK). J. Pain..

[34] Woby S.R., Roach N.K., Urmston M., Watson P.J. (2005). Psychometric Properties of the TSK-11: A Shortened Version of the Tampa Scale for Kinesiophobia. Pain.

[35] Areeudomwong P., Buttagat V. (2017). Reliability and Validity of the Cross-Culturally Adapted Thai Version of the Tampa Scale for Kinesiophobia in Knee Osteoarthritis Patients. Malays. J. Med. Sci..

[36] Gomez P., Sainz de Baranda P., Ortega E., Jordán O., Zafra A. (2014). Design and Validation of a Questionnaire on the Perception of the Athlete Regarding His Return to Training after Injury. Rev. Psicol. Deporte.

[37] Gomez-Piqueras P., Ruiz-Barquín R., Olmedilla A. (2020). Traducción y Adaptación al Inglés de Un Cuestionario Para Determinar La Predisposición Psicológica Del Futbolista Lesionado. Rev. Psicol. Deporte.

[38] Krokos D., Kandanoleon A., Paraskevopoulos E., Tsekoura M., Kapreli E., Christakou A. (2024). Examination of the Validity and Reliability of the Greek Version of the Psychological Readiness of Injured Athlete to Return to Sport (PRIA-RS) Questionnaire. Appl. Sci..

[39] Demirtaş G., Yılmaz Özal Ş., Güzel N.A. (2025). Cross-Cultural Adaptation, Validation, and Reliability Testing of the Psychological Readiness of Injured Athlete to Return to Sport (PRIA-RS) Questionnaire in Turkish Athletes. J. Bodyw. Mov. Ther..

[40] Gómez-Piqueras P., Ardern C., Prieto-Ayuso A., Robles-Palazón F.J., Cejudo A., Sainz de Baranda P., Olmedilla A. (2020). Psychometric Analysis and Effectiveness of the Psychological Readiness of Injured Athlete to Return to Sport (PRIA-RS) Questionnaire on Injured Soccer Players. Int. J. Environ. Res. Public Health.

[41] The Jamovi Project. Jamovi (Version 2.6.26.0). https://www.jamovi.org.

[42] Caglar E., Aşçı F. (2010). Motivational Cluster Profiles of Adolescent Athletes: An Examination of Differences in Physical-Self Perception. J. Sports Sci. Med..

[43] de Miranda Rohlfs I.C.P., Noce F., Wilke C., Terry V.R., Parsons-Smith R.L., Terry P.C. (2024). Prevalence of Specific Mood Profile Clusters among Elite and Youth Athletes at a Brazilian Sports Club. Sports.

[44] Lakicevic N., Roklicer R., Bianco A., Mani D., Paoli A., Trivic T., Ostojic S.M., Milovancev A., Maksimovic N., Drid P. (2020). Effects of Rapid Weight Loss on Judo Athletes: A Systematic Review. Nutrients.

[45] Rouveix M., Bouget M., Pannafieux C., Champely S., Filaire E. (2007). Eating Attitudes, Body Esteem, Perfectionism and Anxiety of Judo Athletes and Nonathletes. Int. J. Sports Med..

[46] Anastasiou K., Morris M., Akam L., Mastana S. (2024). The Genetic Profile of Combat Sport Athletes: A Systematic Review of Physiological, Psychological and Injury Risk Determinants. Int. J. Environ. Res. Public Health.

[47] Mohammadi F. (2019). An Investigation into the Mediation Effect of Coping Style on the Relationship between Psychological Resilience and Perceived Stress among Athletes with Sports Injury. Sport TK Rev. Euroam. Cienc. Deporte.

[48] Quan G., Xiao H., Chen Y. (2025). Exploring the Mechanisms Influencing Psychological Adaptation in Athletes in High-Risk Sports: A Moderated Mediation Model. Sci. Rep..

[49] Osborne R.E., Doty S.A. (2022). Athlete Coping: Personality Dimensions of Recovery from Injury. J. Phys. Educ. Sports Manag..

[50] Baranoff J., Hanrahan S.J., Connor J.P. (2015). The Roles of Acceptance and Catastrophizing in Rehabilitation Following Anterior Cruciate Ligament Reconstruction. J. Sci. Med. Sport..

[51] Sestelo E.S., Ribeiro G.L., Santos C.P.C.d., Goes B.T. (2023). Pain profile and kinesiophobia in judo athletes of the master category. Rev. Pesqui. Em Fisioter..

[52] McNeely L.R., Dudley R.I. (2021). Kinesiophobia Prevalence Among College Athletes. Int. J. Exerc. Sci. Conf. Proc..

[53] Miguez G., Laborda M.A., Miller R.R. (2014). Classical Conditioning and Pain: Conditioned Analgesia and Hyperalgesia. Acta Psychol..

[54] Rescorla R., Wagner A., Black A.H., Prokasy W.F. (1972). A Theory of Pavlovian Conditioning: Variations in the Effectiveness of Reinforcement and Nonreinforcement. Classical Conditioning II: Current Research and Theory.

[55] Ormel J., Jeronimus B.F., Kotov R., Riese H., Bos E.H., Hankin B., Rosmalen J.G.M., Oldehinkel A.J. (2013). Neuroticism and Common Mental Disorders: Meaning and Utility of a Complex Relationship. Clin. Psychol. Rev..

[56] Stilwell P., Harman K. (2019). An Enactive Approach to Pain: Beyond the Biopsychosocial Model. Phenomenol. Cogn. Sci..

[57] Piepiora P.A., Jurczyk J.B., Vveinhardt J. (2025). Mental Preparation of Karateka for Sports Competition in Kata. Front. Sports Act. Living.

